# Supplementary material for: Global Lipidome Profiling Revealed Multifaceted Role of Lipid Species in Hepatitis C Virus Replication, Assembly, and Host Antiviral Response
Source: Viruses. 2023 Feb 7;15(2):464. doi: 10.3390/v15020464 (PMC9965260; doi:10.3390/v15020464)
Supplement: Supplementary file 1 [file viruses-15-00464-s001.zip › viruses-2141735-supplementary.pdf]

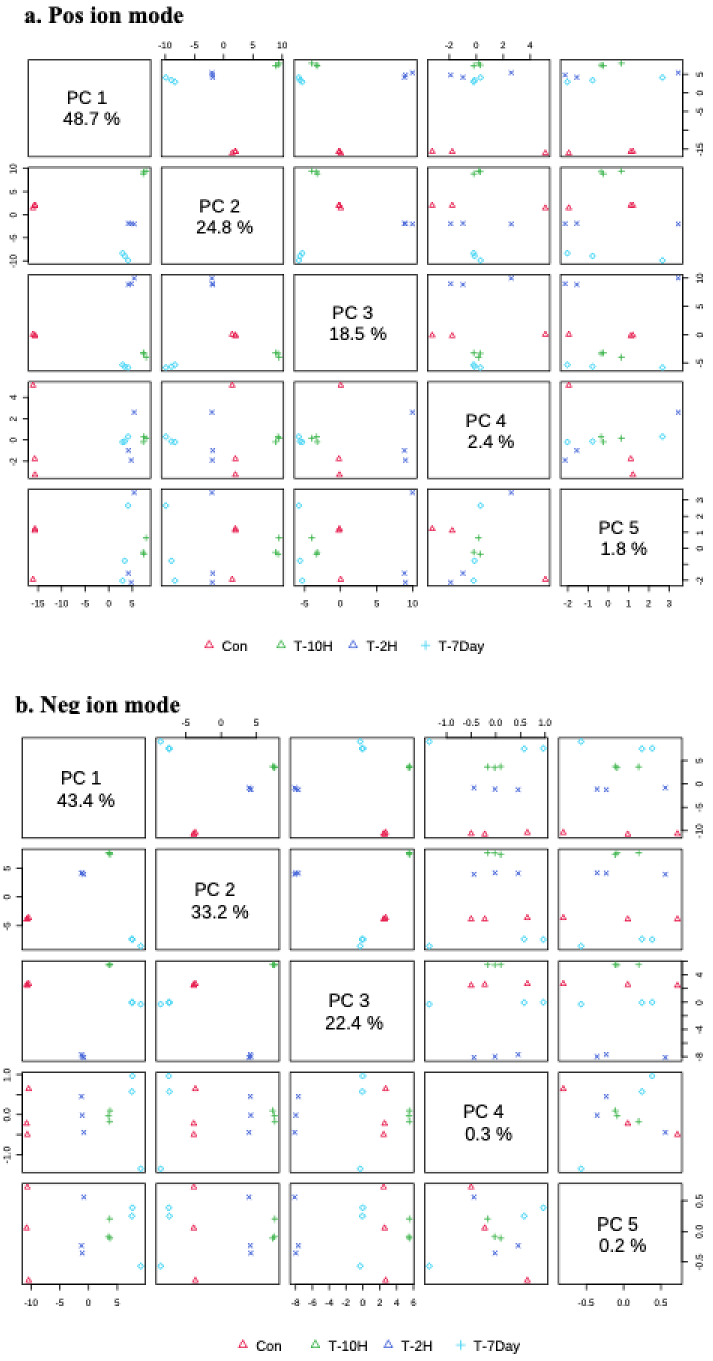

Figure S1. Pairwise score plots between selected Principal components (PC): The explained variance of each PC is shown in the corresponding diagonal cell. Principal component analysis is performed to reduce dimensionality of large data sets. Before performing PCA it is critical to perform standardization by computing variance of different variables. Here different principal components were identified (PC1, PC2, PC and PC4) and their variance was computed (48%, 24%,18.5%,2.4% and 1.8%). Values correspond to each group under study (Red indicates Mock or control, green: 10hr, Blue: 2hr, cyan: Day7). FigureS1a and S1b show the pair wise score plots with corresponding percentage of variance given in diagonal cells. On the basis of variance scores between different PCs, the PCs with highest variance were further used to develop principal component analysis score plot. As depicted in figure S1a, PC1 with the variance score equal to 48.7% and PC2 with variance score of 24.8% were used to develop principal component score plot in

positive ion mode. Similarly in figure S1b, PC1 with variance score equal to 43.4% and PC2 with variance score equal to 33.2% were used to draw a principal component analysis score plot in negative ion mode as shown in figure 3 in main text.

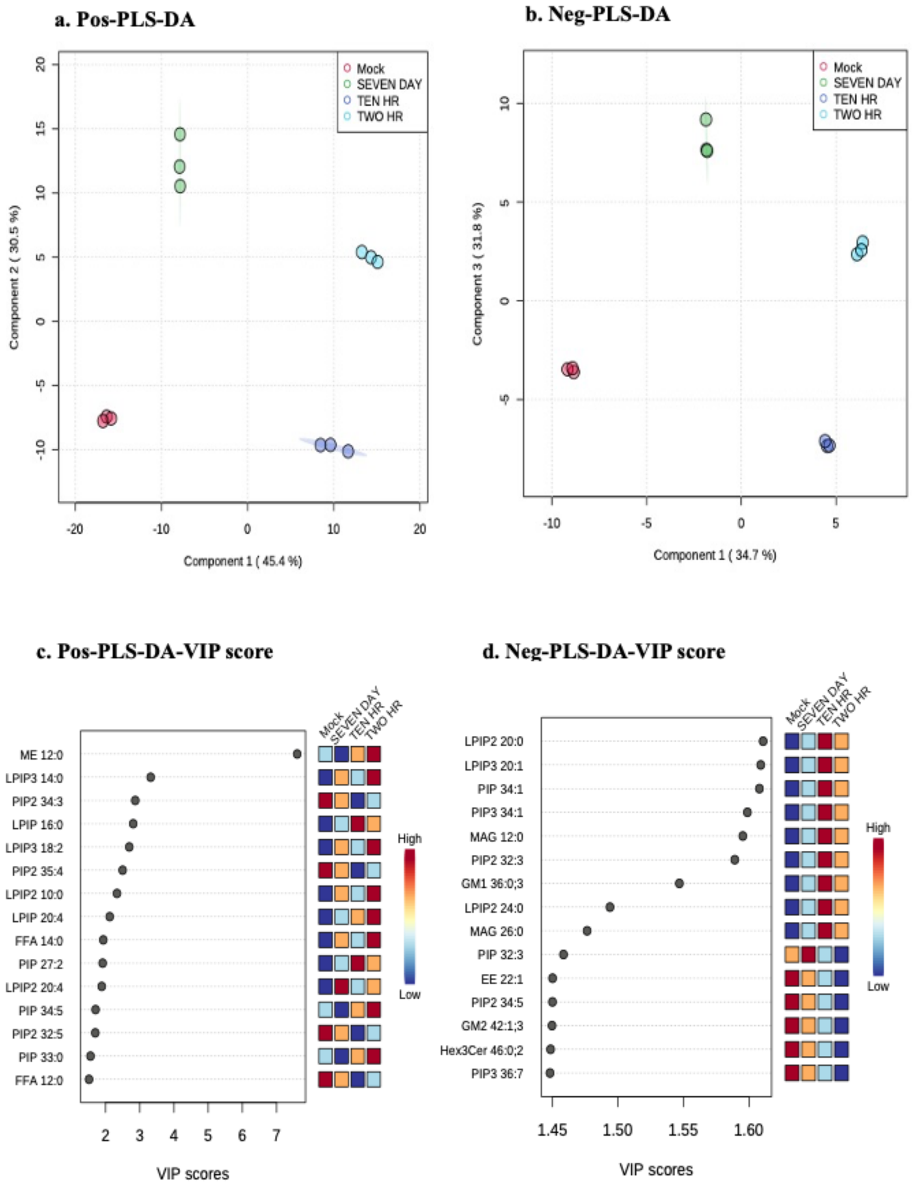

Figure S2: Statistical analysis of lipids in mock- and HCV-infected cells: Partial least squares-discriminant analysis (PLS-DA) represents the versatile algorithm used for discriminative variable selection. it is a supervised version of principal component analysis. Besides its use for dimensional reduction, it can be used for feature selection and classification [66,67]. This method was applied to discriminate between lipid compositions of mock- and HCV-infected Huh7 cells at different time points of HCV infection (2hr, 10hr and Day7). This method facilitated us to investigate the effect of duration of HCV infection on lipidomic profile of cultured Huh7 cells. Figures **S2a and S2b** shows the PLS-DA score plot between principal component 1 and 2 with highest variance score, in positive and negative ion mode respectively. The lipid profile of mock and HCV infected Huh7 cells was clearly classified according to first two principal components with a cumulative contribution rate equal to 73.5% and 67.3% in positive and negative ion mode respectively. VIP scores **(figure S2c and S2d)** of lipids contributing in the differentiation of mock- and HCV-infected samples were estimated, both in positive as well as negative ion mode. The lipid metabolites that are contributing to the classification of control and HCV-infected samples were

identified using VIP scores given in **S2c and S2d**. Lipids having higher VIP scores are more important in contributing to the separation between the various groups, while those with lesser VIP scores have less influence to this model. Here cardinal red indicates highest concentration while as blue indicates the lowest concentration of the metabolites.

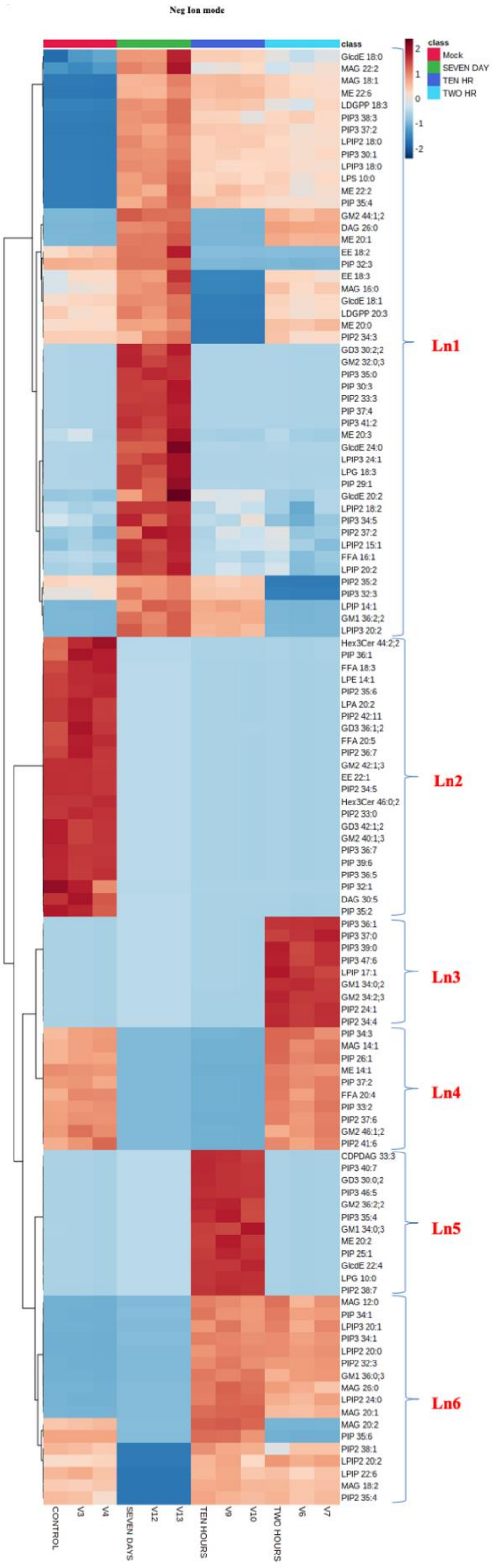

Figure S3: Hierarchical analysis of lipid species: Lipid analytes that have not shown response in positive ion mode were detected in negative ion mode as shown in Figure S3. Based on statistical analysis of lipidomic data from samples under study (Mock, 2hr, 10hr, day7), a heat map was generated which signifies lipid species detected via negative ion mode. The colored bars indicate the level of lipid analyte based on the scale provided where cardinal red indicates highest while as blue indicates lowest concentration of lipid analyte. Here, we show the differential dynamics of lipid species in different samples under study in negative ion mode. To further simplify the analysis, an arbitrary labeling was done as shown in figure S3. Here Ln1 shows the upregulation of lipids at day7 HCV infection, belonging to phosphoinositide like PIP, PIP2 and PIP3, triacyl glycerols (TAGS) that are the precursor molecules for lipid droplets (LDs) formation [68]. Ln3 shows upregulation of PIPs at 2hr of HCV infection and Ln5 shows the upregulation of cDPDAG, GD3, PIP3 and LPG.

Table S1: Top fifty features identified by one-way ANNOVA from Positive Ion. Table S2: Top fifty features identified by one-way ANNOVA: Analysis of variance (ANOVA) was used to evaluate the statistical significance of experimental factors used in our study as reported previously [69]. Tables S1 and S2 represents the top fifty lipid features identified by one way ANOVA in each positive and negative ion mode having p-value less than 0.05. Based on this observation PCA models were prepared and dynamic variability of lipid molecules was accessed in the samples under study.

Table S1. Top fifty features identified by one-way ANNOVA from Positive Ion.

Sample	f. value	p. value	-log10(p)	FDR
**PIP 25:1**	3899.2	5.37E-13	12.27	9.18E-11
**CerCil 46:0;2**	2952.3	1.63E-12	11.787	9.18E-11
**LPIP3 20:1**	2945.4	1.65E-12	11.783	9.18E-11
**PIP2 41:6**	2760.4	2.13E-12	11.671	9.18E-11
**PIP3 33:6**	2424.2	3.59E-12	11.445	1.23E-10
**PIP2 33:0**	2070.2	6.74E-12	11.172	1.81E-10
**PIP 36:5**	2025.5	7.35E-12	11.134	1.81E-10
**GD3 36:0;2**	1801.6	1.17E-11	10.931	2.52E-10
**PIP 34:5**	1578.6	1.99E-11	10.701	3.80E-10
**NAPE 40:4+NH4**	1506.5	2.40E-11	10.62	4.12E-10
**PC 46:1**	1378.9	3.41E-11	10.467	5.34E-10
**LPIP3 14:0**	1290.2	4.45E-11	10.352	6.38E-10
**CerCil 46:2;2**	1062.3	9.66E-11	10.015	1.28E-09
**LPIP 14:0**	946.7	1.53E-10	9.8153	1.77E-09
**PIP2 36:3**	945.05	1.54E-10	9.8122	1.77E-09
**TAG 56:6+NH4**	808.55	2.87E-10	9.5421	3.09E-09
**PIP 30:4**	741.75	4.05E-10	9.3929	4.09E-09
**PIP 27:1**	710.35	4.81E-10	9.3181	4.40E-09
**GD3 36:2;3**	708.42	4.86E-10	9.3134	4.40E-09
**FFA 14:1**	697.44	5.17E-10	9.2863	4.45E-09
**LPA 22:2**	646.77	6.98E-10	9.1559	5.72E-09
**LPE 10:0**	614.7	8.55E-10	9.0679	6.40E-09
**LPIP3 18:2**	614.56	8.56E-10	9.0675	6.40E-09
**LPIP 21:0**	596.66	9.63E-10	9.0165	6.90E-09
**PIP 45:6**	585.35	1.04E-09	8.9834	7.15E-09
**PIP 33:0**	565.64	1.19E-09	8.9242	7.88E-09
**PG 46:2**	542.42	1.41E-09	8.8518	8.96E-09
**TAG 54:0+NH4**	527.52	1.57E-09	8.8036	9.65E-09
**LPIP 20:2**	518.29	1.69E-09	8.7731	9.66E-09
**NAPE 46:3+NH4**	514.35	1.74E-09	8.76	9.66E-09
**NAPE 50:10+NH4**	514.14	1.74E-09	8.7592	9.66E-09
**LPIP3 11:0**	499.23	1.96E-09	8.7084	1.03E-08
**PIP 35:2**	497.56	1.98E-09	8.7026	1.03E-08
**PIP 30:3**	473.54	2.41E-09	8.6172	1.20E-08

**GD1 42:1;2**	472.58	2.43E-09	8.6137	1.20E-08
**TAG 58:9+NH4**	469.21	2.50E-09	8.6013	1.20E-08
**LPIP 20:4**	449.3	2.98E-09	8.5265	1.38E-08
**PIP 36:0**	446.35	3.05E-09	8.5151	1.38E-08
**LPIP2 20:1**	419.72	3.90E-09	8.409	1.69E-08
**PIP2 34:5**	419.04	3.92E-09	8.4062	1.69E-08
**GM2 42:1;2**	395.03	4.96E-09	8.3044	2.03E-08
**GM2 40:1;3**	395.01	4.96E-09	8.3043	2.03E-08
**LMMPE 18:3**	390.91	5.17E-09	8.2863	2.06E-08
**LPIP2 18:3**	389.24	5.26E-09	8.2789	2.06E-08
**LPIP 22:2**	358.91	7.26E-09	8.1391	2.77E-08
**GT3 34:2;2**	337.14	9.30E-09	8.0313	3.42E-08
**PIP2 34:3**	336.66	9.36E-09	8.0289	3.42E-08
**PIP 31:3**	334.59	9.59E-09	8.0182	3.44E-08
**LPIP3 19:0**	320.98	1.13E-08	7.9467	3.97E-08

Table S2: **Top fifty features identified by one-way ANNOVA from Negative ion**

Sample	f.value	p.value	-log10(p)	FDR
**PIP3 36:1**	10659	9.63E-15	14.016	8.50E-13
**PIP3 46:5**	8404.1	2.49E-14	13.604	8.50E-13
**PIP2 34:5**	8071.4	2.93E-14	13.533	8.50E-13
**EE 22:1**	7914	3.17E-14	13.499	8.50E-13
**Hex3Cer 46:0;2**	7681.2	3.57E-14	13.447	8.50E-13
**LPG 10:0**	6823.9	5.73E-14	13.242	1.14E-12
**PIP2 38:7**	6437.2	7.23E-14	13.141	1.23E-12
**GD3 30:0;2**	5690.3	1.18E-13	12.926	1.76E-12
**PIP2 33:0**	4802.1	2.33E-13	12.632	3.09E-12
**GM2 42:1;3**	4599.6	2.77E-13	12.557	3.30E-12
**PIP3 40:7**	3871.8	5.52E-13	12.258	5.97E-12
**GM1 34:0;2**	3388.5	9.41E-13	12.027	9.33E-12
**PIP2 24:1**	2972.5	1.59E-12	11.799	1.45E-11
**PIP 39:6**	2902.6	1.75E-12	11.758	1.48E-11
**CDPDAG 33:3**	2835	1.92E-12	11.717	1.52E-11
**PIP3 36:5**	2762.6	2.13E-12	11.672	1.53E-11
**MAG 20:2**	2742.3	2.19E-12	11.659	1.53E-11
**GlcdE 22:4**	2407.1	3.69E-12	11.433	2.31E-11
**MAG 20:1**	2406.9	3.69E-12	11.433	2.31E-11
**PIP3 36:7**	2317.3	4.29E-12	11.367	2.56E-11
**PIP3 35:0**	2210.3	5.19E-12	11.285	2.94E-11
**PIP2 34:4**	2108.3	6.26E-12	11.203	3.39E-11
**PIP 32:3**	1940	8.73E-12	11.059	4.52E-11
**PIP2 32:3**	1736.7	1.36E-11	10.867	6.74E-11
**PIP 25:1**	1713.6	1.43E-11	10.844	6.82E-11
**PIP 37:4**	1686.8	1.53E-11	10.816	6.99E-11
**GM2 34:2;3**	1615.6	1.81E-11	10.742	7.99E-11
**LPIP2 20:0**	1590.6	1.93E-11	10.714	8.20E-11
**ME 14:1**	1522.9	2.30E-11	10.639	9.42E-11
**MAG 18:2**	1305.1	4.25E-11	10.371	1.69E-10
**LPE 14:1**	1283.4	4.55E-11	10.342	1.74E-10
**PIP3 30:1**	1199	5.96E-11	10.225	2.16E-10
**PIP3 34:1**	1197.1	6.00E-11	10.222	2.16E-10
**PIP2 35:6**	1138.4	7.33E-11	10.135	2.57E-10
**PIP2 42:11**	1056.3	9.89E-11	10.005	3.36E-10
**LPA 20:2**	1017.5	1.15E-10	9.9402	3.79E-10
**PIP 37:2**	981.43	1.33E-10	9.8776	4.26E-10
**GM2 32:0;3**	915.5	1.75E-10	9.7572	5.48E-10
**LPIP2 18:0**	908.88	1.80E-10	9.7447	5.49E-10

**PIP3 37:0**	897.03	1.90E-10	9.7219	5.64E-10
**PIP3 41:2**	881.41	2.03E-10	9.6915	5.91E-10
**PIP 33:2**	858.33	2.26E-10	9.6456	6.41E-10
**PIP 26:1**	844.26	2.42E-10	9.617	6.69E-10
**GD3 42:1;2**	799.33	3.00E-10	9.5223	8.12E-10
**PIP2 37:6**	787.93	3.18E-10	9.4974	8.41E-10
**ME 20:0**	765.93	3.56E-10	9.4484	9.21E-10
**GM1 36:0;3**	755.99	3.75E-10	9.4258	9.50E-10
**GM2 40:1;3**	736.41	4.16E-10	9.3804	1.03E-09
**PIP3 47:6**	726.57	4.39E-10	9.3571	1.07E-09
